# Crystal structure, Hirshfeld surface and frontier mol­ecular orbital analysis of 10-benzyl-9-(3-eth­oxy-4-hy­droxy­phen­yl)-3,3,6,6-tetra­methyl-3,4,6,7,9,10-hexa­hydro­acridine-1,8(2*H*,5*H*)-dione

**DOI:** 10.1107/S2056989020004065

**Published:** 2020-03-27

**Authors:** N. Suresh Babu, V. Sughanya, A. Dhandapani, R. Kalaivanan

**Affiliations:** a Department of Chemistry, Government College of Engineering-Sengipatti, Thanjavur-613 402, Tamil Nadu, India; b Department of Chemistry, Periyar Government Arts College, Silver Beach Road, Devanampattinam, Cuddalore-607 001, Tamil Nadu, India; c Department of Chemistry, CK College of Engineering & Technology, Sellankuppam, Cuddalore-607 003, Tamil Nadu, India; d Department of Chemistry, Annamalai University, Annamalai Nagar-608 002, Tamil Nadu, India

**Keywords:** crystal structure, dimedone, benzyl­amine, acridinedione

## Abstract

In the fused ring system of the title compound, C_32_H_37_NO_4_, the central di­hydro­pyridine ring adopts a flattened boat conformation, the mean and maximum deviations of the di­hydro­pyridine ring being 0.1429 (2) and 0.2621 (2) Å, respectively. The two cyclo­hexenone rings adopt envelope conformations with the tetra­substituted C atoms as flap atoms. The benzene and phenyl rings form dihedral angles of 85.81 (2) and 88.90 (2)°, respectively, with the mean plane of the di­hydro­pyridine ring. In the crystal, mol­ecules are linked *via* an O—H⋯O hydrogen bond, forming a helical chain along the *b-*axis direction. A Hirshfeld surface analysis indicates that the most important contributions to the crystal packing are from H⋯H (65.2%), O⋯H/H⋯O (18.8%) and C⋯H/H⋯C (13.9%) contacts. Quantum chemical calculations for the frontier mol­ecular orbitals were undertake to determine the chemical reactivity of the title compound.

## Abstract 2

In the acridinedione moiety of the title compound, the central ring adopts a flattened-boat conformation, whereas the cyclo­hexenone rings adopt envelope conformations.

## Chemical context   

The crystal structures of acridinedione derivatives are expected to provide useful information on the mol­ecular conformation, which has a direct relationship to biological activity. Acridine derivatives (Nasim & Brychcy, 1979 ▸; Thull & Testa, 1994 ▸; Mándi *et al.*, 1994 ▸), well known as therapeutic agents, are important because of their range of applications in the dye and pharmaceutical industries. Certain acridinedione derivatives exhibit good inhibition against the pathogen vibro isolate-I (Josephrajan *et al.*, 2005 ▸), display anti-cancer (Sondhi *et al.*, 2004 ▸; Sugaya *et al.*, 1994 ▸; Kimura *et al.*, 1993 ▸) and anti­tumour (Talacki *et al.*, 1974 ▸) activity and act as K-channel openers (Li *et al.*, 1996 ▸).
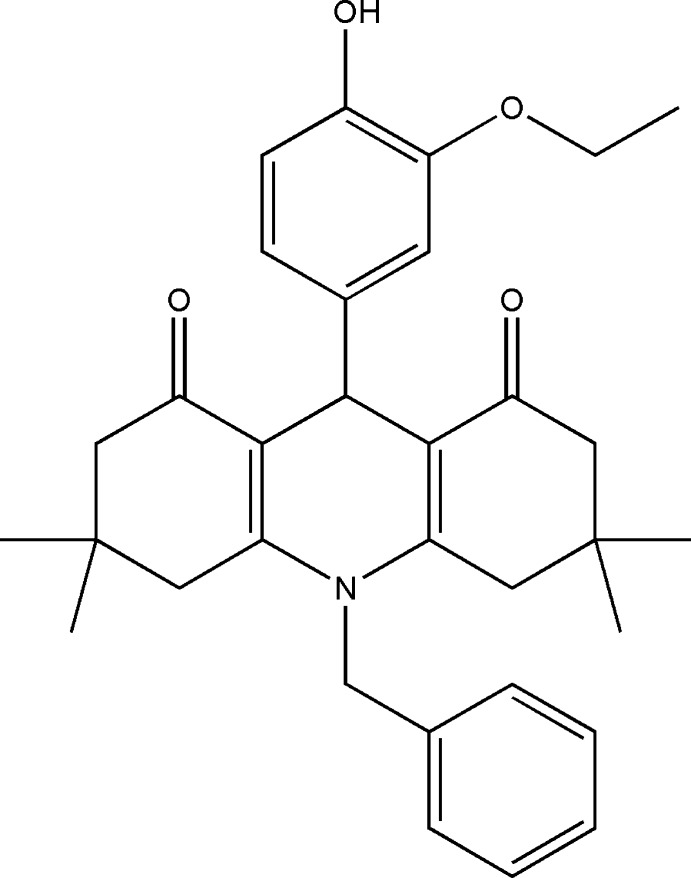


## Structural commentary   

**Figure 1:**
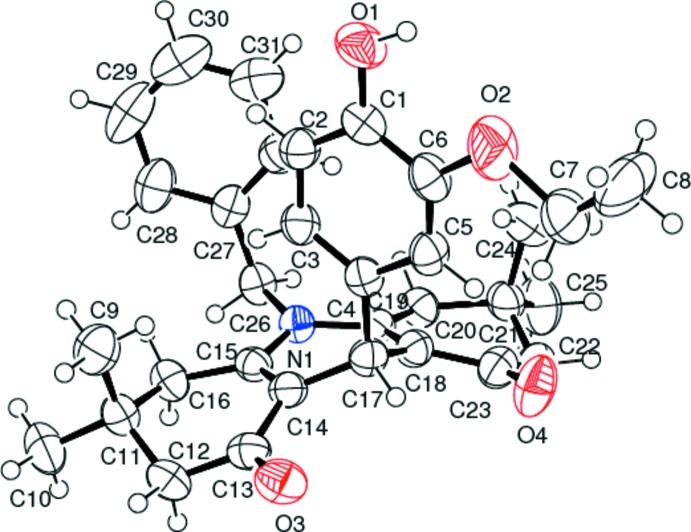
The mol­ecular structure of the title compound, showing the atom-numbering scheme. Displacement ellipsoids are drawn at the 50% probability level. Only one component of the disordered ethyl group is shown.

The rings *A* (C18–C23), *B* (N1/C15/C14/C17–C19) and *C* (C11–C16) in the fused-ring system show total puckering amplitudes *Q*(*T*) of 0.4624 (2), 0.3888 (2) and 0.4942 (3) Å, respectively. The central ring *B* adopts a flattened boat conformation with a mean deviation of 0.1429 (2) Å from the mean plane and a maximum deviation of 0.2621 (2) Å for atom C17. The cyclo­hexenone rings *A* and *C* adopt envelope conformations with atoms C21 and C11 as the respective flap atoms, being situated out of the mean plane of each ring by 0.3084 (2) and 0.3341 (2) Å (Fig. 1 ▸). The puckering parameters are φ = 202.98 (2)° and θ = 58.16 (2)° for *A*, φ = −1.87 (9)° and θ = 107.81 (3)° for *B*, and φ =17.95 (6)° and θ = 62.30° for *C*. The benzene (C1–C6) and phenyl (C27–C32) rings form dihedral angles of 85.81 (2) and 88.90 (2)°, respectively, with the di­hydro­pyridine mean plane. In the di­hydro­pyridine ring, the lengths of the C14=C15 and C18=C19 double bonds are 1.356 (3) and 1.354 (3) Å, respectively. The C15—C14—C13 [119.70 (19)]° and C19—C18—C23 [121.0 (2)°] angles are almost the same. The ethyl group is disordered over two sites with occupancies of 0.572 (11) and 0.428 (11).

## Frontier mol­ecular orbital analysis   

**Figure 2:**
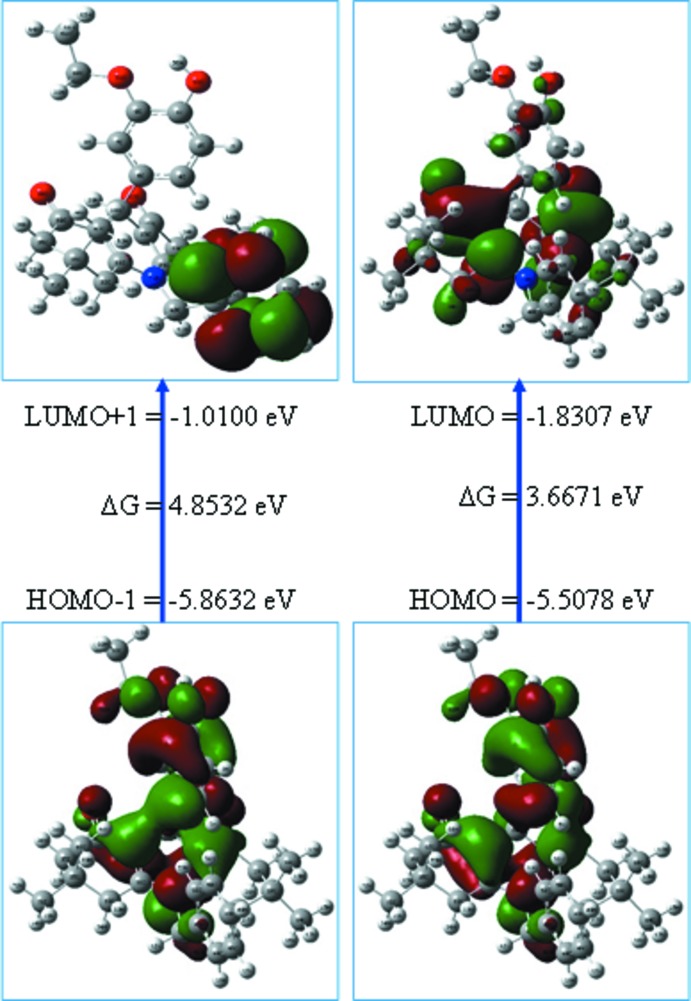
The frontier mol­ecular orbitals of the title compound, showing positive (red) and negative (green) regions.

The chemical reactivity of the title compound was studied by frontier mol­ecular orbital analysis. For the calculation, the starting structural geometry was taken from the refined experimental structure obtained from X-ray diffraction data. The energy levels for the compound were computed using the DFT-B3LYP/6-311G++(d,p) level of theory as implemented in *Gaussian09W* (Frisch *et al.*, 2010 ▸). The calculated frontier mol­ecular orbitals, HOMO-1, HOMO, LUMO and LUMO+1, are shown in Fig. 2 ▸. The energies of HOMO-1, HOMO, LUMO and LUMO+1 were calculated to be −5.8632, −5.5078, −1.8307 and −1.0100 eV, respectively, and the energy required to excite one electron from HOMO to LUMO and from HOMO-1 to LUMO+1 are 3.6671 and 4.8532 eV, respectively. The chemical potential, chemical hardness, chemical softness and electrophilicity index of the title mol­ecule are listed in Table 1 ▸. Parr *et al.* (1999 ▸) have proposed the electrophilicity index as a qu­anti­tative measure of the energy lowering due to the maximal electron flow between donor and acceptor orbitals. The electrophilicity index value of 3.6714 eV shows the global electrophilic nature of the mol­ecule. Based on the wide band gap and its chemical hardness value of 1.8335 eV, the title mol­ecule seems to be hard.

## Supra­molecular features and Hirshfeld surface analysis   

**Figure 3:**
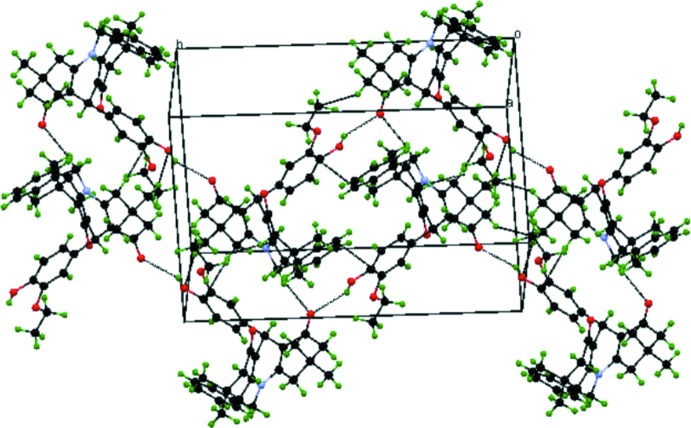
A packing diagram of the title compound, showing the O—H⋯O and C—H⋯O hydrogen bonds (dashed lines).

In the crystal, the mol­ecules are linked *via* O1—H1⋯O3^i^ hydrogen bonds, forming helical chains along the *b*-axis direction (Table 2 ▸). The chains are further connected by weak C26—H26*B*⋯O3^ii^ hydrogen bonds, forming a sheet structure parallel to (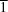
01) (Fig. 3 ▸).

**Figure 4:**
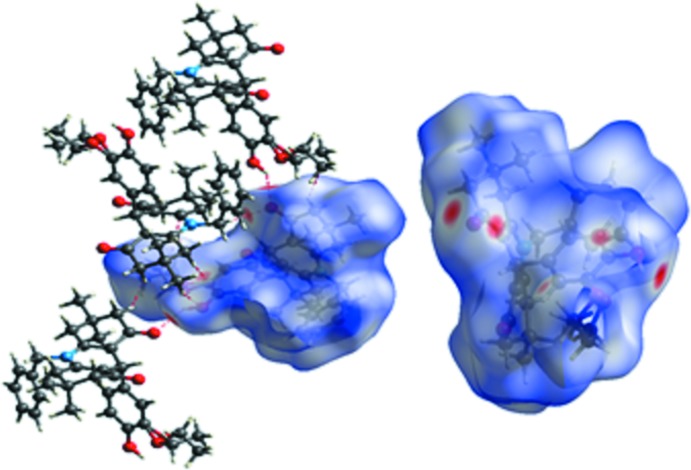
Hirshfeld surfaces of the title compound mapped over *d*_norm_.

**Figure 5:**
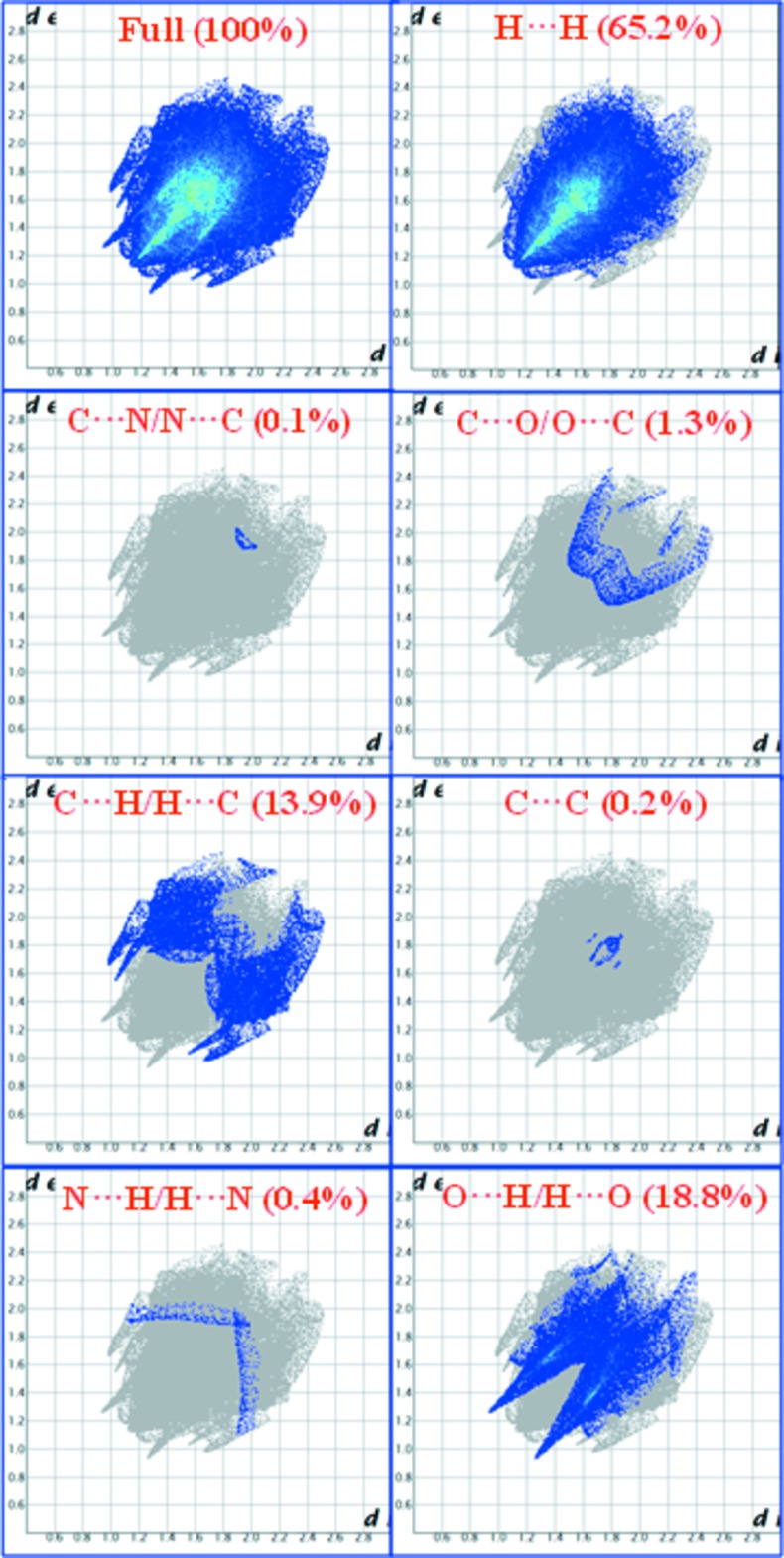
Two-dimensional fingerprint plots for the title compound.

To qu­antify the inter­molecular contacts in the crystal, Hirshfeld surfaces (Spackman & Jayatilaka, 2009 ▸) and two-dimensional fingerprint plots were generated using *Crystal Explorer 3.1* (Wolff *et al.*, 2012 ▸). The Hirshfeld surfaces mapped over *d*_norm_ (Fig. 4 ▸) show the inter­molecular contacts as red-coloured spots, which indicate the closer contacts of C—H⋯O and O—H⋯O hydrogen bonds. The 2D fingerprint plots are illustrated in Fig. 5 ▸. The H⋯H contacts comprise 65.2% of the total inter­actions. Besides these contacts, O⋯H/H⋯O (18.8%) and C⋯H/H⋯C (13.9%) inter­actions make a significant contribution to the total Hirshfeld surface. The percentage contributions of the C⋯N/N⋯C, C⋯O/O⋯C, N⋯H/H⋯N and C⋯C contacts are 0.1, 1.3, 0.4 and 0.2%, respectively.

## Database survey   

The bond lengths in the title compound, are close to those reported for similar compounds, for example, 10-benzyl-9-(3,4-di­meth­oxy­phen­yl)-3,3,6,6-tetra­methyl-3,4,6,7,9,10-hexa­hydro­acridine-1,8(2*H*,5*H*)-dione (Sureshbabu & Sughanya, 2015 ▸) and 10-benzyl-9-(4-eth­oxy­phen­yl)-3,3,6,6-tetra­methyl-3,4,6,7,9,10-hexa­hydro­acridine-1,8(2*H*,5*H*)-dione (Sughanya & Sureshbabu, 2012 ▸).

## Synthesis and crystallization   

A mixture of 3-eth­oxy-4-hy­droxy­benzaldehyde (0.498 g, 3 mmol), 5,5-di­methyl­cyclo­hexane-1,3-dione (0.84 g, 6 mmol) and benzyl­amine (0.33 g, 3 mmol) was dissolved in 30 ml of acetic acid. The solution was refluxed for 6 h with the reaction being monitored by TLC. When the reaction was complete, the reaction mixture was poured into ice-cold water and stirred well. The formed precipitate was filtered and dried. Yellowsingle crystals suitable for X-ray diffraction were obtained from an ethanol solution at room temperature. (m.p. 471 K, 1.30 g, 2.6 mmol, yield 86%). IR (KBr): cm^−1^ 3427, 2958, 1634, 1559, 1513, 1430, 1376, 1275, 1240, 1202, 1120, 1041, 966. ^1^H NMR (400 MHz, CDCl_3_): *δ* 0.89 (*s*, 6H), 0.99 (*s*, 6H), 1.39 (*t*, 3H), 2.20 (*s*, 4H), 2.39 (*dd*, 4H), 4.89 (*s*, 2H), 5.23 (*s*, 1H), 6.55 (*d*, 1H), 6.69 (*d*, 1H), 7.06 (*s*, 1H), 7.16 (*d*, 2H), 7.41–7.34 (*m*, 3H). ^13^C NMR (75 MHz, CDCl_3_): *δ* 14.89, 28.07&28.63, 40.27, 50.06, 64.29, 112.79–150.22, 195.77.

## Refinement   

Crystal data, data collection and structure refinement details are summarized in Table 3 ▸. C-bound H atoms were fixed in calculated positions (C—H = 0.93–0.98 Å) and allowed to ride with respect to the parent atoms with *U*_iso_(H) = 1.2 or 1.5*U*_eq_(C). The O-bound H atom was refined freely. For the disordered ethyl group, bond distance and displacement restraints (*DFIX*, *SADI* and *SIMU*) were applied.

## Supplementary Material

Crystal structure: contains datablock(s) global, I. DOI: 10.1107/S2056989020004065/is5533sup1.cif

Structure factors: contains datablock(s) I. DOI: 10.1107/S2056989020004065/is5533Isup2.hkl

RES file. DOI: 10.1107/S2056989020004065/is5533sup3.txt

Figure 1S. Hirshfeld surfaces of the title compound, mapped over de, di, shape index and curvedness. DOI: 10.1107/S2056989020004065/is5533sup4.pdf

CCDC reference: 924670

Additional supporting information:  crystallographic information; 3D view; checkCIF report

## Figures and Tables

**Table 1:** The global reactivity descriptors of the title compound

Frontier mol­ecular orbitals	Energy
*E*_HOMO_	−5.5078
*E*_LUMO_	−1.8307
*E*_HOMO_−1	−5.8632
*E*_LUMO_+1	−1.0100
(*E*_HOMO_ and *E*_LUMO_) gap	3.6671
(*E*_HOMO_−1 and *E*_LUMO_+1) gap	4.8532
Chemical potential (μ)	3.6692
Chemical hardness (η)	1.8335
Chemical softness (*S*)	0.5454
Electrophilicity index (ω)	3.6714

**Table 2:** Hydrogen-bond geometry (Å, °)

*D*—H⋯*A*	*D*—H	H⋯*A*	*D*⋯*A*	*D*—H⋯*A*
O1—H1⋯O3^i^	0.85 (3)	2.23 (4)	2.893 (2)	135 (3)
C26—H26*B*⋯O3^ii^	0.97	2.40	3.258 (3)	148

Symmetry codes: (i) 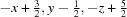; (ii) 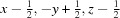.

**Table 3:** Experimental details

Crystal data	
Chemical formula	C_32_H_37_NO_4_
*M*_r_	499.62
Crystal system, space group	Monoclinic, *P*2_1_/*n*
Temperature (K)	296
*a*, *b*, *c* (Å)	10.5780 (2), 18.4190 (5), 14.3980 (3)
β (°)	108.791 (1)
*V* (Å^3^)	2655.73 (10)
*Z*	4
Radiation type	Mo *K*α
μ (mm^−1^)	0.08
Crystal size (mm)	0.35 × 0.30 × 0.30

Data collection	
Diffractometer	Bruker Kappa APEXII
Absorption correction	Multi-scan (*SADABS*; Bruker, 2004 ▸)
*T*_min_, *T*_max_	0.674, 0.746
No. of measured, independent and observed [*I* > 2σ(*I*)] reflections	23101, 4681, 3374
*R*_int_	0.034
(sin θ/λ)_max_ (Å^−1^)	0.595

Refinement	
*R*[*F* ^2^ > 2σ(*F* ^2^)], *wR*(*F* ^2^), *S*	0.049, 0.139, 1.02
No. of reflections	4681
No. of parameters	364
No. of restraints	39
H-atom treatment	H atoms treated by a mixture of independent and constrained refinement
Δρ_max_, Δρ_min_ (e Å^−3^)	0.37, −0.24

Computer programs: *APEX3*, *SAINT* and *XPREP* (Bruker, 2004 ▸), *SIR92* (Altomare *et al.*, 1993 ▸), *SHELXL2018* (Sheldrick, 2015 ▸), *ORTEP-3 for Windows* (Farrugia, 2012 ▸) and *Mercury* (Macrae *et al.*, 2020 ▸).

## supplementary crystallographic information

### Crystal data

**Table:**

C_32_H_37_NO_4_	*F*(000) = 1072
*M**_r_* = 499.62	*D*_x_ = 1.250 Mg m^−^^3^
Monoclinic, *P*2_1_/*n*	Mo *K*α radiation, λ = 0.71073 Å
*a* = 10.5780 (2) Å	Cell parameters from 5176 reflections
*b* = 18.4190 (5) Å	θ = 2.1–23.3°
*c* = 14.3980 (3) Å	µ = 0.08 mm^−^^1^
β = 108.791 (1)°	*T* = 296 K
*V* = 2655.73 (10) Å^3^	Block, yellow
*Z* = 4	0.35 × 0.30 × 0.30 mm

### Data collection

**Table:**

Bruker Kappa APEXII diffractometer	4681 independent reflections
Radiation source: fine-focus sealed tube	3374 reflections with *I* > 2σ(*I*)
Graphite monochromator	*R*_int_ = 0.034
ω and φ scan	θ_max_ = 25.0°, θ_min_ = 1.9°
Absorption correction: multi-scan (SADABS; Bruker, 2004)	*h* = −12→12
*T*_min_ = 0.674, *T*_max_ = 0.746	*k* = −21→21
23101 measured reflections	*l* = −17→17

### Refinement

**Table:**

Refinement on *F*^2^	Hydrogen site location: mixed
Least-squares matrix: full	H atoms treated by a mixture of independent and constrained refinement
*R*[*F*^2^ > 2σ(*F*^2^)] = 0.049	*w* = 1/[σ^2^(*F*_o_^2^) + (0.0564*P*)^2^ + 1.304*P*] where *P* = (*F*_o_^2^ + 2*F*_c_^2^)/3
*wR*(*F*^2^) = 0.139	(Δ/σ)_max_ = 0.003
*S* = 1.02	Δρ_max_ = 0.37 e Å^−^^3^
4681 reflections	Δρ_min_ = −0.24 e Å^−^^3^
364 parameters	Extinction correction: SHELXL2018 (Sheldrick, 2015), Fc^*^=kFc[1+0.001xFc^2^λ^3^/sin(2θ)]^-1/4^
39 restraints	Extinction coefficient: 0.0066 (9)

### Special details

**Table:**

Geometry. All esds (except the esd in the dihedral angle between two l.s. planes) are estimated using the full covariance matrix. The cell esds are taken into account individually in the estimation of esds in distances, angles and torsion angles; correlations between esds in cell parameters are only used when they are defined by crystal symmetry. An approximate (isotropic) treatment of cell esds is used for estimating esds involving l.s. planes.

### Fractional atomic coordinates and isotropic or equivalent isotropic displacement parameters (Å^2^)

**Table:**

	*x*	*y*	*z*	*U*_iso_*/*U*_eq_	Occ. (<1)
C1	0.7372 (2)	0.07235 (11)	1.19469 (15)	0.0471 (5)	
C2	0.7273 (2)	0.10618 (12)	1.10816 (15)	0.0488 (5)	
H2	0.777437	0.089182	1.070203	0.059*	
C3	0.6443 (2)	0.16503 (12)	1.07625 (15)	0.0461 (5)	
H3	0.640119	0.187332	1.017378	0.055*	
C4	0.5675 (2)	0.19150 (11)	1.12980 (14)	0.0407 (5)	
C5	0.5779 (2)	0.15710 (12)	1.21800 (15)	0.0485 (5)	
H5	0.527092	0.173864	1.255627	0.058*	
C6	0.6615 (2)	0.09894 (13)	1.25058 (15)	0.0525 (6)	
C9	0.7670 (2)	0.33092 (16)	0.9497 (2)	0.0704 (7)	
H9A	0.754428	0.297468	0.997122	0.106*	
H9B	0.841043	0.362289	0.980882	0.106*	
H9C	0.785001	0.304417	0.897943	0.106*	
C10	0.6623 (3)	0.42747 (15)	0.8297 (2)	0.0741 (8)	
H10A	0.676549	0.399414	0.777748	0.111*	
H10B	0.738811	0.457558	0.859168	0.111*	
H10C	0.584716	0.457489	0.803512	0.111*	
C11	0.6411 (2)	0.37629 (12)	0.90727 (17)	0.0524 (6)	
C12	0.6105 (3)	0.41915 (12)	0.98762 (19)	0.0604 (6)	
H12A	0.687205	0.448978	1.021231	0.072*	
H12B	0.535806	0.451328	0.957972	0.072*	
C13	0.5775 (2)	0.37160 (11)	1.06128 (16)	0.0465 (5)	
C14	0.5053 (2)	0.30484 (10)	1.02566 (15)	0.0416 (5)	
C15	0.46639 (19)	0.28917 (10)	0.92851 (14)	0.0397 (5)	
C16	0.5213 (2)	0.32806 (12)	0.85821 (16)	0.0492 (5)	
H16A	0.451065	0.357700	0.814830	0.059*	
H16B	0.547020	0.292318	0.818172	0.059*	
C17	0.4695 (2)	0.25444 (11)	1.09529 (14)	0.0426 (5)	
H17	0.468205	0.282359	1.152911	0.051*	
C18	0.3300 (2)	0.22705 (11)	1.04337 (15)	0.0432 (5)	
C19	0.29089 (19)	0.21317 (11)	0.94584 (15)	0.0399 (5)	
C20	0.1604 (2)	0.17691 (12)	0.89287 (16)	0.0473 (5)	
H20A	0.176648	0.138375	0.852218	0.057*	
H20B	0.102079	0.212117	0.849634	0.057*	
C21	0.0881 (2)	0.14485 (12)	0.95949 (17)	0.0509 (5)	
C22	0.0967 (2)	0.19754 (14)	1.04238 (19)	0.0624 (7)	
H22A	0.047079	0.241160	1.015377	0.075*	
H22B	0.055315	0.175713	1.086706	0.075*	
C23	0.2381 (2)	0.21791 (13)	1.09917 (18)	0.0548 (6)	
C26	0.3491 (2)	0.20767 (12)	0.79067 (14)	0.0455 (5)	
H26A	0.357805	0.246910	0.748202	0.055*	
H26B	0.259352	0.188286	0.764455	0.055*	
C27	0.4480 (2)	0.14911 (12)	0.79111 (14)	0.0457 (5)	
C28	0.5291 (3)	0.15385 (17)	0.73326 (18)	0.0697 (7)	
H28	0.522216	0.193597	0.692092	0.084*	
C29	0.6213 (3)	0.0992 (2)	0.7363 (2)	0.0898 (10)	
H29	0.676908	0.102922	0.697950	0.108*	
C30	0.6304 (3)	0.0403 (2)	0.7953 (2)	0.0856 (9)	
H30	0.691243	0.003581	0.796294	0.103*	
C31	0.5509 (3)	0.03490 (15)	0.8525 (2)	0.0720 (7)	
H31	0.557448	−0.005257	0.892928	0.086*	
C32	0.4609 (2)	0.08898 (13)	0.85044 (18)	0.0568 (6)	
H32	0.407100	0.084969	0.890121	0.068*	
N1	0.37004 (16)	0.23636 (9)	0.89039 (11)	0.0398 (4)	
O1	0.82089 (18)	0.01441 (9)	1.22322 (14)	0.0647 (5)	
O3	0.60779 (17)	0.39009 (9)	1.14761 (12)	0.0612 (5)	
O4	0.27281 (18)	0.22766 (12)	1.18772 (13)	0.0788 (6)	
O2	0.6794 (2)	0.06291 (12)	1.33650 (13)	0.0898 (7)	
C7	0.5937 (8)	0.0989 (5)	1.3972 (5)	0.079 (2)	0.572 (11)
H7A	0.611903	0.150541	1.405357	0.095*	0.572 (11)
H7B	0.499043	0.092146	1.363367	0.095*	0.572 (11)
C8	0.6318 (9)	0.0638 (6)	1.4895 (5)	0.131 (4)	0.572 (11)
H8A	0.580043	0.082713	1.527912	0.197*	0.572 (11)
H8B	0.724873	0.072391	1.523026	0.197*	0.572 (11)
H8C	0.616247	0.012594	1.480136	0.197*	0.572 (11)
C7'	0.5829 (9)	0.0565 (6)	1.3908 (6)	0.082 (3)	0.428 (11)
H7'1	0.493748	0.073595	1.354927	0.099*	0.428 (11)
H7'2	0.580760	0.009096	1.419461	0.099*	0.428 (11)
C8'	0.6622 (12)	0.1071 (5)	1.4551 (10)	0.142 (6)	0.428 (11)
H8'1	0.621787	0.119635	1.503820	0.213*	0.428 (11)
H8'2	0.670497	0.149885	1.419379	0.213*	0.428 (11)
H8'3	0.749065	0.086785	1.486481	0.213*	0.428 (11)
C24	0.1545 (3)	0.07364 (14)	1.0030 (2)	0.0769 (8)	
H24A	0.247265	0.082123	1.037911	0.115*	
H24B	0.146600	0.039373	0.951142	0.115*	
H24C	0.111638	0.054559	1.047206	0.115*	
C25	−0.0558 (3)	0.13003 (19)	0.8986 (2)	0.0835 (9)	
H25A	−0.102569	0.109844	0.939704	0.125*	
H25B	−0.058132	0.096260	0.847274	0.125*	
H25C	−0.097909	0.174597	0.870218	0.125*	
H1	0.814 (3)	−0.0069 (19)	1.274 (2)	0.107 (12)*	

### Atomic displacement parameters (Å^2^)

**Table:**

	*U*^11^	*U*^22^	*U*^33^	*U*^12^	*U*^13^	*U*^23^
C1	0.0481 (12)	0.0434 (12)	0.0435 (12)	−0.0042 (10)	0.0057 (10)	0.0027 (9)
C2	0.0544 (13)	0.0500 (13)	0.0443 (12)	0.0038 (10)	0.0191 (10)	0.0019 (10)
C3	0.0534 (13)	0.0499 (12)	0.0357 (11)	0.0010 (10)	0.0152 (10)	0.0048 (9)
C4	0.0436 (11)	0.0414 (11)	0.0349 (10)	−0.0094 (9)	0.0095 (9)	−0.0062 (8)
C5	0.0573 (13)	0.0536 (13)	0.0368 (11)	−0.0063 (11)	0.0182 (10)	−0.0047 (10)
C6	0.0629 (14)	0.0573 (14)	0.0346 (11)	−0.0069 (11)	0.0117 (10)	0.0063 (10)
C9	0.0469 (14)	0.0768 (18)	0.0881 (19)	0.0009 (13)	0.0225 (13)	0.0029 (15)
C10	0.0785 (18)	0.0664 (17)	0.0857 (19)	−0.0175 (14)	0.0380 (16)	0.0090 (14)
C11	0.0513 (13)	0.0459 (12)	0.0629 (14)	−0.0066 (10)	0.0225 (11)	0.0020 (11)
C12	0.0676 (16)	0.0403 (12)	0.0767 (16)	−0.0118 (11)	0.0279 (13)	−0.0050 (11)
C13	0.0421 (12)	0.0409 (11)	0.0535 (13)	0.0006 (9)	0.0112 (10)	−0.0068 (10)
C14	0.0402 (11)	0.0378 (11)	0.0467 (12)	0.0005 (9)	0.0140 (9)	−0.0024 (9)
C15	0.0364 (10)	0.0361 (10)	0.0446 (11)	0.0019 (8)	0.0103 (9)	0.0011 (9)
C16	0.0499 (13)	0.0480 (12)	0.0483 (12)	−0.0032 (10)	0.0140 (10)	0.0068 (10)
C17	0.0466 (12)	0.0435 (11)	0.0382 (11)	−0.0045 (9)	0.0143 (9)	−0.0085 (9)
C18	0.0434 (11)	0.0431 (12)	0.0443 (12)	−0.0004 (9)	0.0155 (9)	−0.0025 (9)
C19	0.0362 (11)	0.0382 (11)	0.0456 (11)	0.0011 (8)	0.0135 (9)	0.0010 (9)
C20	0.0399 (11)	0.0487 (12)	0.0500 (12)	−0.0035 (10)	0.0097 (10)	0.0016 (10)
C21	0.0437 (12)	0.0470 (12)	0.0629 (14)	−0.0055 (10)	0.0184 (11)	0.0014 (11)
C22	0.0540 (14)	0.0650 (16)	0.0768 (17)	−0.0006 (12)	0.0329 (13)	0.0002 (13)
C23	0.0576 (14)	0.0559 (14)	0.0565 (14)	−0.0013 (11)	0.0263 (12)	−0.0049 (11)
C26	0.0463 (12)	0.0533 (13)	0.0338 (10)	−0.0077 (10)	0.0085 (9)	0.0000 (9)
C27	0.0403 (11)	0.0582 (13)	0.0357 (11)	−0.0093 (10)	0.0084 (9)	−0.0125 (10)
C28	0.0666 (16)	0.096 (2)	0.0527 (14)	−0.0061 (15)	0.0276 (13)	−0.0071 (14)
C29	0.0633 (18)	0.141 (3)	0.073 (2)	0.006 (2)	0.0337 (16)	−0.026 (2)
C30	0.0634 (18)	0.103 (2)	0.083 (2)	0.0173 (17)	0.0123 (16)	−0.0294 (19)
C31	0.0641 (16)	0.0656 (17)	0.0790 (19)	0.0055 (13)	0.0127 (15)	−0.0123 (14)
C32	0.0535 (14)	0.0560 (14)	0.0615 (15)	−0.0020 (11)	0.0191 (12)	−0.0089 (12)
N1	0.0391 (9)	0.0421 (9)	0.0372 (9)	−0.0040 (7)	0.0108 (7)	−0.0021 (7)
O1	0.0711 (11)	0.0564 (10)	0.0623 (11)	0.0109 (9)	0.0154 (9)	0.0187 (9)
O3	0.0687 (11)	0.0526 (10)	0.0549 (10)	−0.0101 (8)	0.0097 (8)	−0.0138 (8)
O4	0.0754 (12)	0.1155 (16)	0.0554 (11)	−0.0146 (11)	0.0348 (9)	−0.0161 (10)
O2	0.1117 (16)	0.1106 (16)	0.0534 (11)	0.0161 (13)	0.0353 (11)	0.0358 (11)
C7	0.091 (5)	0.082 (5)	0.070 (4)	0.018 (5)	0.036 (3)	0.029 (4)
C8	0.182 (7)	0.154 (8)	0.090 (5)	0.087 (6)	0.091 (5)	0.041 (4)
C7'	0.084 (5)	0.059 (5)	0.084 (6)	−0.018 (4)	−0.002 (4)	0.027 (5)
C8'	0.138 (9)	0.073 (6)	0.136 (9)	−0.032 (6)	−0.066 (7)	0.034 (6)
C24	0.091 (2)	0.0520 (15)	0.090 (2)	−0.0023 (14)	0.0310 (17)	0.0113 (14)
C25	0.0545 (16)	0.103 (2)	0.091 (2)	−0.0266 (15)	0.0210 (15)	−0.0013 (18)

### Geometric parameters (Å, º)

**Table:**

C1—O1	1.363 (3)	C21—C22	1.518 (3)
C1—C2	1.367 (3)	C21—C24	1.524 (3)
C1—C6	1.394 (3)	C22—C23	1.503 (3)
C2—C3	1.377 (3)	C22—H22A	0.9700
C2—H2	0.9300	C22—H22B	0.9700
C3—C4	1.377 (3)	C23—O4	1.221 (3)
C3—H3	0.9300	C26—N1	1.478 (2)
C4—C5	1.391 (3)	C26—C27	1.501 (3)
C4—C17	1.527 (3)	C26—H26A	0.9700
C5—C6	1.372 (3)	C26—H26B	0.9700
C5—H5	0.9300	C27—C28	1.377 (3)
C6—O2	1.362 (3)	C27—C32	1.378 (3)
C9—C11	1.524 (3)	C28—C29	1.393 (4)
C9—H9A	0.9600	C28—H28	0.9300
C9—H9B	0.9600	C29—C30	1.362 (5)
C9—H9C	0.9600	C29—H29	0.9300
C10—C11	1.532 (3)	C30—C31	1.357 (4)
C10—H10A	0.9600	C30—H30	0.9300
C10—H10B	0.9600	C31—C32	1.372 (3)
C10—H10C	0.9600	C31—H31	0.9300
C11—C12	1.519 (3)	C32—H32	0.9300
C11—C16	1.522 (3)	O1—H1	0.85 (3)
C12—C13	1.501 (3)	O2—C7'	1.476 (9)
C12—H12A	0.9700	O2—C7	1.592 (6)
C12—H12B	0.9700	C7—C8	1.414 (10)
C13—O3	1.228 (3)	C7—H7A	0.9700
C13—C14	1.451 (3)	C7—H7B	0.9700
C14—C15	1.356 (3)	C8—H8A	0.9600
C14—C17	1.502 (3)	C8—H8B	0.9600
C15—N1	1.388 (2)	C8—H8C	0.9600
C15—C16	1.501 (3)	C7'—C8'	1.389 (12)
C16—H16A	0.9700	C7'—H7'1	0.9700
C16—H16B	0.9700	C7'—H7'2	0.9700
C17—C18	1.510 (3)	C8'—H8'1	0.9600
C17—H17	0.9800	C8'—H8'2	0.9600
C18—C19	1.354 (3)	C8'—H8'3	0.9600
C18—C23	1.456 (3)	C24—H24A	0.9600
C19—N1	1.397 (2)	C24—H24B	0.9600
C19—C20	1.502 (3)	C24—H24C	0.9600
C20—C21	1.525 (3)	C25—H25A	0.9600
C20—H20A	0.9700	C25—H25B	0.9600
C20—H20B	0.9700	C25—H25C	0.9600
C21—C25	1.517 (3)		
			
O1—C1—C2	118.9 (2)	C25—C21—C20	108.6 (2)
O1—C1—C6	122.6 (2)	C22—C21—C20	109.37 (18)
C2—C1—C6	118.5 (2)	C24—C21—C20	109.46 (19)
C1—C2—C3	121.1 (2)	C23—C22—C21	112.62 (19)
C1—C2—H2	119.5	C23—C22—H22A	109.1
C3—C2—H2	119.5	C21—C22—H22A	109.1
C2—C3—C4	121.29 (19)	C23—C22—H22B	109.1
C2—C3—H3	119.4	C21—C22—H22B	109.1
C4—C3—H3	119.4	H22A—C22—H22B	107.8
C3—C4—C5	117.5 (2)	O4—C23—C18	122.0 (2)
C3—C4—C17	123.20 (18)	O4—C23—C22	121.1 (2)
C5—C4—C17	119.22 (18)	C18—C23—C22	116.9 (2)
C6—C5—C4	121.3 (2)	N1—C26—C27	111.52 (16)
C6—C5—H5	119.3	N1—C26—H26A	109.3
C4—C5—H5	119.3	C27—C26—H26A	109.3
O2—C6—C5	125.2 (2)	N1—C26—H26B	109.3
O2—C6—C1	114.6 (2)	C27—C26—H26B	109.3
C5—C6—C1	120.26 (19)	H26A—C26—H26B	108.0
C11—C9—H9A	109.5	C28—C27—C32	118.0 (2)
C11—C9—H9B	109.5	C28—C27—C26	121.4 (2)
H9A—C9—H9B	109.5	C32—C27—C26	120.63 (19)
C11—C9—H9C	109.5	C27—C28—C29	120.1 (3)
H9A—C9—H9C	109.5	C27—C28—H28	120.0
H9B—C9—H9C	109.5	C29—C28—H28	120.0
C11—C10—H10A	109.5	C30—C29—C28	120.2 (3)
C11—C10—H10B	109.5	C30—C29—H29	119.9
H10A—C10—H10B	109.5	C28—C29—H29	119.9
C11—C10—H10C	109.5	C31—C30—C29	120.2 (3)
H10A—C10—H10C	109.5	C31—C30—H30	119.9
H10B—C10—H10C	109.5	C29—C30—H30	119.9
C12—C11—C16	107.89 (18)	C30—C31—C32	119.7 (3)
C12—C11—C9	110.6 (2)	C30—C31—H31	120.1
C16—C11—C9	110.84 (19)	C32—C31—H31	120.1
C12—C11—C10	110.6 (2)	C31—C32—C27	121.7 (2)
C16—C11—C10	108.4 (2)	C31—C32—H32	119.1
C9—C11—C10	108.4 (2)	C27—C32—H32	119.1
C13—C12—C11	112.95 (18)	C15—N1—C19	119.16 (16)
C13—C12—H12A	109.0	C15—N1—C26	119.72 (16)
C11—C12—H12A	109.0	C19—N1—C26	121.07 (16)
C13—C12—H12B	109.0	C1—O1—H1	113 (2)
C11—C12—H12B	109.0	C6—O2—C7'	127.0 (4)
H12A—C12—H12B	107.8	C6—O2—C7	111.0 (3)
O3—C13—C14	121.9 (2)	C8—C7—O2	106.6 (5)
O3—C13—C12	120.6 (2)	C8—C7—H7A	110.4
C14—C13—C12	117.41 (19)	O2—C7—H7A	110.4
C15—C14—C13	119.70 (19)	C8—C7—H7B	110.4
C15—C14—C17	119.93 (18)	O2—C7—H7B	110.4
C13—C14—C17	120.33 (18)	H7A—C7—H7B	108.6
C14—C15—N1	119.84 (18)	C7—C8—H8A	109.5
C14—C15—C16	122.63 (18)	C7—C8—H8B	109.5
N1—C15—C16	117.52 (17)	H8A—C8—H8B	109.5
C15—C16—C11	114.24 (18)	C7—C8—H8C	109.5
C15—C16—H16A	108.7	H8A—C8—H8C	109.5
C11—C16—H16A	108.7	H8B—C8—H8C	109.5
C15—C16—H16B	108.7	C8'—C7'—O2	85.9 (9)
C11—C16—H16B	108.7	C8'—C7'—H7'1	114.3
H16A—C16—H16B	107.6	O2—C7'—H7'1	114.3
C14—C17—C18	107.01 (16)	C8'—C7'—H7'2	114.3
C14—C17—C4	113.31 (16)	O2—C7'—H7'2	114.3
C18—C17—C4	111.10 (16)	H7'1—C7'—H7'2	111.5
C14—C17—H17	108.4	C7'—C8'—H8'1	109.5
C18—C17—H17	108.4	C7'—C8'—H8'2	109.5
C4—C17—H17	108.4	H8'1—C8'—H8'2	109.5
C19—C18—C23	121.0 (2)	C7'—C8'—H8'3	109.5
C19—C18—C17	119.97 (18)	H8'1—C8'—H8'3	109.5
C23—C18—C17	119.00 (18)	H8'2—C8'—H8'3	109.5
C18—C19—N1	119.73 (18)	C21—C24—H24A	109.5
C18—C19—C20	122.28 (18)	C21—C24—H24B	109.5
N1—C19—C20	117.90 (17)	H24A—C24—H24B	109.5
C19—C20—C21	114.70 (18)	C21—C24—H24C	109.5
C19—C20—H20A	108.6	H24A—C24—H24C	109.5
C21—C20—H20A	108.6	H24B—C24—H24C	109.5
C19—C20—H20B	108.6	C21—C25—H25A	109.5
C21—C20—H20B	108.6	C21—C25—H25B	109.5
H20A—C20—H20B	107.6	H25A—C25—H25B	109.5
C25—C21—C22	111.4 (2)	C21—C25—H25C	109.5
C25—C21—C24	109.1 (2)	H25A—C25—H25C	109.5
C22—C21—C24	108.9 (2)	H25B—C25—H25C	109.5
			
O1—C1—C2—C3	−179.9 (2)	C17—C18—C19—N1	−11.6 (3)
C6—C1—C2—C3	−0.3 (3)	C23—C18—C19—C20	−9.8 (3)
C1—C2—C3—C4	−0.6 (3)	C17—C18—C19—C20	171.90 (18)
C2—C3—C4—C5	0.7 (3)	C18—C19—C20—C21	−10.6 (3)
C2—C3—C4—C17	−177.22 (19)	N1—C19—C20—C21	172.77 (18)
C3—C4—C5—C6	0.0 (3)	C19—C20—C21—C25	164.0 (2)
C17—C4—C5—C6	178.02 (19)	C19—C20—C21—C22	42.2 (3)
C4—C5—C6—O2	178.9 (2)	C19—C20—C21—C24	−77.1 (2)
C4—C5—C6—C1	−0.9 (3)	C25—C21—C22—C23	−175.4 (2)
O1—C1—C6—O2	0.8 (3)	C24—C21—C22—C23	64.2 (3)
C2—C1—C6—O2	−178.8 (2)	C20—C21—C22—C23	−55.4 (3)
O1—C1—C6—C5	−179.5 (2)	C19—C18—C23—O4	177.4 (2)
C2—C1—C6—C5	1.0 (3)	C17—C18—C23—O4	−4.3 (3)
C16—C11—C12—C13	56.7 (3)	C19—C18—C23—C22	−4.1 (3)
C9—C11—C12—C13	−64.7 (3)	C17—C18—C23—C22	174.2 (2)
C10—C11—C12—C13	175.2 (2)	C21—C22—C23—O4	−143.8 (2)
C11—C12—C13—O3	147.3 (2)	C21—C22—C23—C18	37.6 (3)
C11—C12—C13—C14	−34.7 (3)	N1—C26—C27—C28	−124.2 (2)
O3—C13—C14—C15	175.6 (2)	N1—C26—C27—C32	55.3 (3)
C12—C13—C14—C15	−2.4 (3)	C32—C27—C28—C29	−0.5 (4)
O3—C13—C14—C17	−2.3 (3)	C26—C27—C28—C29	179.0 (2)
C12—C13—C14—C17	179.70 (19)	C27—C28—C29—C30	1.0 (4)
C13—C14—C15—N1	−163.65 (18)	C28—C29—C30—C31	−0.9 (5)
C17—C14—C15—N1	14.2 (3)	C29—C30—C31—C32	0.3 (4)
C13—C14—C15—C16	14.9 (3)	C30—C31—C32—C27	0.3 (4)
C17—C14—C15—C16	−167.17 (18)	C28—C27—C32—C31	−0.2 (3)
C14—C15—C16—C11	10.5 (3)	C26—C27—C32—C31	−179.7 (2)
N1—C15—C16—C11	−170.87 (18)	C14—C15—N1—C19	16.0 (3)
C12—C11—C16—C15	−44.9 (2)	C16—C15—N1—C19	−162.68 (18)
C9—C11—C16—C15	76.4 (2)	C14—C15—N1—C26	−166.38 (18)
C10—C11—C16—C15	−164.7 (2)	C16—C15—N1—C26	15.0 (3)
C15—C14—C17—C18	−38.2 (2)	C18—C19—N1—C15	−17.3 (3)
C13—C14—C17—C18	139.68 (18)	C20—C19—N1—C15	159.41 (18)
C15—C14—C17—C4	84.6 (2)	C18—C19—N1—C26	165.12 (18)
C13—C14—C17—C4	−97.5 (2)	C20—C19—N1—C26	−18.2 (3)
C3—C4—C17—C14	−25.6 (3)	C27—C26—N1—C15	82.8 (2)
C5—C4—C17—C14	156.47 (18)	C27—C26—N1—C19	−99.7 (2)
C3—C4—C17—C18	94.9 (2)	C5—C6—O2—C7'	26.6 (6)
C5—C4—C17—C18	−83.0 (2)	C1—C6—O2—C7'	−153.6 (5)
C14—C17—C18—C19	36.8 (2)	C5—C6—O2—C7	−2.0 (5)
C4—C17—C18—C19	−87.4 (2)	C1—C6—O2—C7	177.7 (4)
C14—C17—C18—C23	−141.50 (19)	C6—O2—C7—C8	−172.7 (8)
C4—C17—C18—C23	94.3 (2)	C6—O2—C7'—C8'	−104.5 (6)
C23—C18—C19—N1	166.72 (19)		

### Hydrogen-bond geometry (Å, º)

**Table:**

*D*—H···*A*	*D*—H	H···*A*	*D*···*A*	*D*—H···*A*
O1—H1···O3^i^	0.85 (3)	2.23 (4)	2.893 (2)	135 (3)
C26—H26*B*···O3^ii^	0.97	2.40	3.258 (3)	148

Symmetry codes: (i) −*x*+3/2, *y*−1/2, −*z*+5/2; (ii) *x*−1/2, −*y*+1/2, *z*−1/2.
