# Supplementary figures and images for: Crystal structure, Hirshfeld surface and frontier mol­ecular orbital analysis of 10-benzyl-9-(3-eth­oxy-4-hy­droxy­phen­yl)-3,3,6,6-tetra­methyl-3,4,6,7,9,10-hexa­hydro­acridine-1,8(2H,5H)-dione
Source: Acta Crystallogr E Crystallogr Commun. 2020 Mar 27;76(Pt 4):585–8. doi: 10.1107/S2056989020004065 (PMC7133029; doi:10.1107/S2056989020004065)

Figure 1S

Hirshfeld surfaces of the title compound, mapped over  $d_e$ ,  $d_i$ , shape index and curvedness.

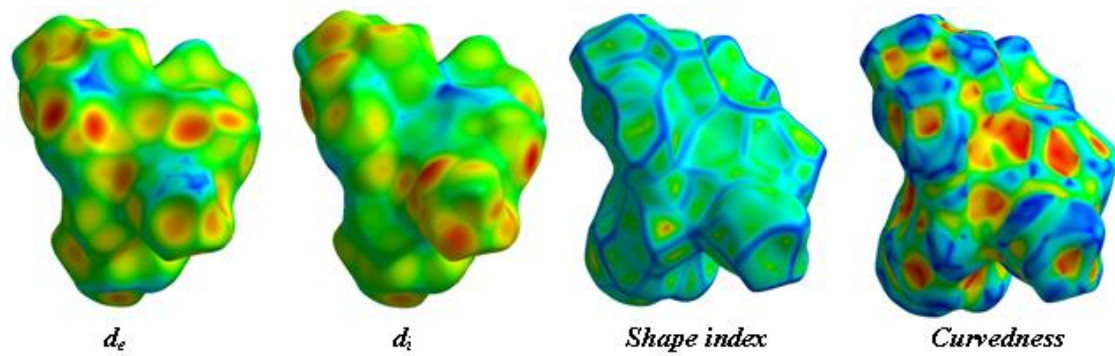

Supplement: Supplementary file 4 [file e-76-00585-sup4.pdf]
